# Does Leisure Boredom Predict Short Video Addiction in Adolescents?

**DOI:** 10.1007/s11126-025-10172-4

**Published:** 2025-06-12

**Authors:** Önder Baltacı, Işılay Açar

**Affiliations:** 1 Faculty of Education, Department of Psychological Counseling, Kırşehir Ahi Evran University, Kırşehir, Turkey; 2 Institute of Social Sciences, Department of Psychological Counseling, Kırşehir Ahi Evran University, Kırşehir, Turkey

**Keywords:** Leisure Boredom, Depression, Sensation Seeking, Short Video Addiction, Adolescents

## Abstract

Leisure boredom during adolescence is a psychological state that can lead to various maladaptive behaviors. This state is linked to increased emotional distress and a higher risk of problematic behaviors, particularly short video addiction. The aim of this study was to examine the multiple mediating role of depression and sensation seeking in the relationship between leisure boredom and short video addiction. The study was conducted with 361 adolescents (62.3% female, 37.7% male), with ages ranging from 14 to 19 years (*M*_*age*_ = 16.11, *SD* = 1.41). The data of this research was analysed using structural equation modeling. The findings of the study indicate that the relationship between leisure boredom and short video addiction was fully mediated by two variables (depression and sensation seeking). Specifically, adolescents experiencing leisure boredom were more likely to report higher levels of depression and sensation seeking, leading to a higher risk of short video addiction. The results suggest that reducing depressive symptoms and regulating sensation seeking behavior may be effective in preventing short video addiction among adolescents experiencing boredom during leisure time. Overall, these findings emphasize the importance of mental health support and engagement strategies in leisure time to mitigate the negative impact of boredom on short video addiction in adolescents. The findings of the study were discussed and interpreted in detail in the light of the relevant literature.

**Supplementary Information:**

The online version contains supplementary material available at 10.1007/s11126-025-10172-4.

## Introduction

The rapid development of social media platforms has significantly transformed the way individuals consume digital content. In this process of change, short video applications—characterized by rapidly consumable and stimulating content—have come to the forefront (Ye et al. 2025). Recently, the use of short video platforms such as TikTok, Instagram Reels, and YouTube Shorts has become increasingly widespread, particularly emerging as a digital entertainment environment among adolescents (Chen et al. 2024). These platforms specifically offer fast-paced and engaging content targeted at younger audiences (Wang et al. 2023). Adolescents, due to their ongoing identity development, heightened need for peer affiliation, and limited self-regulatory capacity, may be particularly vulnerable to such content (Nesi et al. 2020). These developmental factors help explain why short video platforms are especially appealing for this age group. Recent global reports emphasize a substantial rise in the use of short video applications among adolescents. A study conducted by the Pew Research Center (2023) revealed that approximately 67% of American teenagers reported using TikTok, and nearly 15% indicated using the platform “almost constantly.” Similarly, a report by Common Sense Media (2022) found that screen time dedicated to entertainment media—including short video platforms—has sharply increased since the COVID-19 pandemic, with adolescents spending an average of 1.5 to 2 h per day exclusively on short video content.

Although these platforms are often perceived as harmless sources of entertainment, the excessive and widespread use of short video platforms among adolescents has raised concerns about the risk of addiction (Ding et al. 2024). Unlike traditional media content, the interactive and immersive nature of short videos may increase their addictive potential among adolescents (Xie et al. 2023). Existing literature suggests that the excessive, uncontrolled use of short video content—especially when it disrupts social functioning—may lead to the development of short video addiction (Türk and Yıldırım 2024). While short video addiction, characterized by symptoms such as loss of control, preoccupation, mood changes, and withdrawal, shares similarities with other behavioral addictions, it remains an understudied phenomenon (Zhang et al. 2019a, b). This phenomenon, although not yet formally recognized in diagnostic classification systems such as the DSM-5 or ICD-11, is increasingly discussed within the domain of behavioral addictions (Montag et al. 2021). Research on the impact of short video addiction among adolescents highlights a range of negative consequences, including those related to mental health, social behavior, and academic performance (Ming et al. 2023; Xu et al. 2023; J.-H. Ye et al. 2022; Yu et al. 2024). Considering the psychosocial developmental characteristics of adolescence, it is essential to examine the underlying mechanisms of short video addiction during this period. Identifying the variables that influence short video addiction is of critical importance for developing effective prevention and intervention strategies (Guo and Chai 2024). Building on this foundation, this study investigates both direct and mediated pathways to deepen the understanding of the mechanisms underlying short video addiction. Specifically, it explores how leisure boredom directly impacts short video addiction while also examining its indirect effects through depression and sensation seeking.

## Leisure Boredom and Short Video Addiction

Leisure boredom, defined as the perceived lack of meaningful, satisfying, or engaging activities during free time, can be a significant predictor of various maladaptive behaviors among adolescents (Isacescu et al. 2017). This concept arises when there is a mismatch between an individual’s need for optimal stimulation and the quality or availability of leisure opportunities (Eastwood et al. 2012). According to Arousal Theory (Zuckerman 2014), insufficient stimulation—particularly during unstructured free time—may lead to boredom, restlessness, or compensatory behaviors. Developmental factors such as adolescents’ desire for novelty, pursuit of autonomy, and underdeveloped self-regulation can place them at greater risk for experiencing leisure boredom (Elhai et al. 2017). This may lead adolescents to turn to digital media as a way to escape boredom (Lepp et al. 2017; Wegmann et al. 2018). Indeed, the Compensatory Internet Use Theory (Kardefelt-Winther 2014) posits that individuals often seek online environments to cope with negative emotions in their lives. In this context, the accessibility of short video platforms, combined with algorithmic structures that offer constant novelty and gratification, may position these platforms as a form of escape for adolescents experiencing leisure boredom (Zhang et al. 2019a, b). Over time, such usage may become excessive and uncontrolled, thereby increasing the risk of short video addiction (Xie et al. 2023). Previous research has shown that individuals with a higher tendency toward leisure boredom tend to spend more time on short video applications (Lepp et al. 2025; Wolniewicz et al. 2020; Y. Zhang et al. 2024a, b). As a result, leisure boredom may increase adolescents’ risk of developing short video addiction. Beyond this direct effect, other variables may allow for a deeper understanding of the relationship between leisure boredom and short video addiction. In this context, this study examines depression and sensation seeking as potential mediating variables.

### Depression as a Mediator

During adolescence—a period marked by emotional sensitivity, identity development, and an increased need for stimulation—leisure boredom is increasingly recognized as a risk factor for adolescents’ well-being (Spruyt et al. 2020). Adolescents experiencing leisure boredom may struggle with concerns about leading a meaningful life (Bälter et al. 2023). According to Self-Determination Theory, unstructured and unsatisfying leisure time may hinder the fulfillment of three basic psychological needs—autonomy, competence, and relatedness—thereby contributing to experiences of leisure boredom (Ryan and Deci 2017). This situation may render adolescents more vulnerable to emotional difficulties such as depression. Frustrating these basic needs is known to undermine well-being and increase vulnerability to depressive symptoms (Vansteenkiste and Ryan 2013). This theoretical perspective is supported by empirical research showing that individuals experiencing leisure boredom report higher levels of depressive symptoms (Hager et al. 2022; Lee and Zelman 2019). Importantly, depression is one of the most common mental health problems in adolescence, with global prevalence estimates ranging from 10 to 20% (World Health Organization [WHO], 2021). This makes it a highly relevant construct in studies focusing on adolescent behavioral risk. Moreover, elevated depressive symptoms may lead adolescents to turn more frequently to short video platforms as a form of escapism (Zhang et al. 2024a, b). The Compensatory Internet Use Theory (Kardefelt-Winther 2014) posits that individuals may engage more with digital media tools as a way of coping with negative experiences in their lives. In this regard, short video platforms become particularly appealing to adolescents with depressive symptoms by diverting their attention and providing short-term mood shifts. Additionally, adolescents with depressive symptoms may spend increased time on short video platforms in an attempt to avoid negative emotions, which in turn heightens the risk of developing an addiction (Chao et al. 2023). Prior research indicates that depression is a significant predictor of excessive short video platform use (Yu et al. 2024; Zhang et al. 2024a, b). Therefore, it can be said that depression may serve as a mediating factor in the relationship between leisure boredom and short video addiction.

### Sensation Seeking as a Mediator

Sensation seeking may be a potential mediating variable in the relationship between leisure boredom and short video addiction. Leisure boredom may increase sensation seeking tendencies among adolescents (Motamedi et al. 2020). When adolescents perceive their spare time as lacking in stimulation and meaning and experience boredom, they may be more inclined to engage in sensation seeking behaviors—defined as the pursuit of novel, varied, and intense experiences (X.-C. Zhang et al. 2022). The Optimal Arousal Theory (Zuckerman 2014) emphasizes that individuals strive to maintain a certain level of internal arousal, and when stimulation falls below this threshold, the motivation to engage in more stimulating behaviors increases. In this context, adolescents experiencing leisure boredom may seek excitement as a way to cope with their under-stimulated state (Freund et al. 2021). Although limited, research suggests that high levels of leisure boredom may lead to increased sensation seeking behavior (Gordon and Caltabiano 1996; Lepp et al. 2025). An elevated need for sensation may, in turn, lead to increased time spent on short video platforms. Indeed, individuals with high sensation seeking tendencies are more drawn to short videos, which offer novelty, intensity, and rapid feedback (Choi and Cho 2019). Applications like TikTok and Instagram Reels provide a stream of unpredictable and highly stimulating content, making them particularly appealing to adolescents with high sensation seeking traits (Huang et al. 2022). The way this content rewards users can make the brain’s pleasure and impulsivity pathways stronger, which might raise the chances of becoming addicted. Existing studies support this perspective, highlighting strong associations between sensation seeking and technology-related addictive behaviors (Meng et al. 2024; Tian et al. 2019; Wang et al. 2019, 2020). Therefore, sensation seeking may mediate the relationship between leisure boredom and short video addiction.

## The Present Study

Despite growing concerns about the excessive use of short video platforms among adolescents, relatively few studies have examined the mechanisms contributing to short video addiction in this population (Jiang and Yoo 2024; Zhang et al. 2024a, b; Zhu et al. 2024). Behavioral addictions such as short video addiction are known to pose a risk to adolescents’ well-being (Chao et al. 2023). Short video addiction has been associated with academic underachievement, sleep disturbances, social isolation, and psychological distress (Jiang and Yoo 2024; Xie et al. 2023; J.-H. Ye et al. 2022). During adolescence—a critical period for psychosocial development—the highly stimulating nature of short video content may hinder emotional well-being (Xie et al. 2023). In this regard, identifying the variables that contribute to short video addiction is crucial for developing effective prevention and intervention strategies. These studies can also offer a theoretical foundation for comprehending the wider consequences of short video addiction. Within this framework, the current study explores the multiple mediating roles of depression and sensation seeking in the relationship between leisure boredom and short video addiction. Consequently, the study posits the following hypotheses:

### H_1_

Leisure boredom significantly predicts short video addiction.

### H_2_

Depression mediates the relationship between leisure boredom and short video addiction.

### H_3_

Sensation seeking mediates the relationship between leisure boredom and short video addiction.

## Method

### Participant and Procedure

This study, which used convenience sampling methods, involved a total of 361 adolescents, 225 female (62.3%) and 136 male (37.7%). The participants ranged in age from 14 to 19, and their mean age was 16.11 years (SD = 1.41 years). In terms of the number of social media accounts, 147 adolescents (40.7%) had accounts in 1 or 2 social media applications, while 127 adolescents (35.2%) had accounts in 3 or 4 social media applications. In terms of daily social media usage time, 173 adolescents (47.9%) spent 2–3 h and 123 adolescents (34.1%) spent more than 4 h on social media.

During the research procedure, the ethical rules stated in the Declaration of Helsinki were adhered to throughout the research. Professional ethical principles were adhered to throughout the research. Research data were collected face to face from participants based on the principle of voluntariness and both informed consent from the adolescent participants and parental consent were obtained before participation. The study did not pay any fees to the participants.

### Measures

#### ***Leisure Boredom Scale***

The scale was developed by (Iso-Ahola and Weissinger 1990) to assess subjective perceptions of leisure boredom. The Turkish adaptation of the scale was carried out by (Kara et al. 2014). The scale is a ten-item scale (e.g., ‘I waste too much of my leisure time sleeping.’) that assesses self-rated leisure boredom. Items are rated on a 5-point scale from 1 (absolutely disagree) to 5 (absolutely agree). Items 3, 4, 5, 8, and 9 are reverse-scored in the scale. The scale comprises two sub-dimensions: boredom, and satisfaction. Higher scores on the scale indicate the higher the perception of boredom in leisure time. The Turkish version of the scale exhibits robust psychometric properties, including very good construct validity (x^2^/df = 1.83, RMSEA = 0.05, SRMR = 0.05, CFI = 0.95, GFI = 0.96, NFI = 0.90), and good reliability (α = 0.72).

#### ***Kutcher Adolescent Depression Scale– Short Form***

The scale was developed by (LeBlanc et al. 2002) to determine the depression levels of adolescents. The Turkish adaptation of the scale was carried out by (Tatar and Bekiroğlu 2019). The scale is a six-item scale (e.g., ‘Feeling worried, nervous, agitated, tense, anxious.’) that assesses self-rated adolescents depression. Items are rated on a 4-point scale from 0 (almost never) to 3 (always). Higher scores on the scale indicate greater levels of adolescent depression. The scale comprises a single dimension. The Turkish version of the scale exhibits robust psychometric properties, including very good construct validity (x^2^/df = 5.52, RMSEA = 0.05, SRMR = 0.02, CFI = 0.99, GFI = 0.99, AGFI = 0.98, NFI = 0.99), and excellent reliability (α = 0.82).

#### ***Brief Sensation Seeking Scale***

The scale was developed by (Stephenson et al. 2003) to determine the sensation seeking levels of adolescents. The Turkish adaptation of the scale was carried out by (Çelik 2015). The scale is a four-item scale (e.g., ‘I like to do scary things.’) that assesses self-rated adolescents sensation seeking behaviors. Items are rated on a 4-point scale from 1 (strongly disagree) to 4 (strongly agree). Higher scores on the scale indicate greater levels of adolescent sensation seeking. The scale comprises a single dimension. The Turkish version of the scale exhibits robust psychometric properties, including very good construct validity (x^2^ = 523,376; *p* < 0.001, KMO = 0.75), and excellent reliability (α = 0.81).

#### ***Short Video Addiction Scale***

The scale was developed by (J.-H. Ye et al. 2022) to determine the short video addiction levels of adolescents. The Turkish adaptation of the scale was carried out by (Türk and Yıldırım 2024). The scale is a ten-item scale (e.g., ‘Others complain or criticize me for my habit of watching short videos.’) that assesses self-rated adolescents short video addiction. Items are rated on a 5-point scale from 1 (strongly disagree) to 5 (strongly agree). Higher scores on the scale indicate greater levels of adolescent short video addiction. The scale comprises a single dimension. The Turkish version of the scale exhibits robust psychometric properties, including very good construct validity (x^2^/df = 3.33, RMSEA = 0.12, SRMR = 0.08, CFI = 0.86, GFI = 0.88, AGFI = 0.80, IFI = 0.86), and excellent reliability (α = 0.82).

### Data Analysis

In this study, SPSS 25.0 was used for descriptive statistics and correlation analysis, and AMOS Graphics 24.0 statistical program for structural equation modeling. In this study, firstly the normality test of the data was examined. After ensuring normal distribution, descriptive statistics and correlation coefficients were calculated. Then, two-stage structural equation modeling suggested by (Anderson and Gerbing 1988) was conducted to test the hypotheses of the study. The first of these two stages is to examine the measurement model, and the second is to test the structural model. In both models, the fit index values are examined to determine the fit between the model and the data. The literature states that the values of CFI, TLI, NFI, GFI, and IFI for these fit index values should exceed 0.90, while the values of SRMR and RMSEA should be less than 0.08 (Hu and Bentler 1999). The multiple mediation model was tested using 5,000 bootstrap samples. The significance of the direct and indirect effects was assessed based on 95% bias-corrected bootstrap confidence intervals. An effect was considered statistically significant if the confidence interval did not include zero. In the analyses of this study, the item parceling technique was used for unidimensional constructs. Specifically, two parcels were created for each of the depression and short video addiction variables. This technique is often preferred to reduce measurement error and improve model estimation in SEM, particularly when applied to scales with confirmed unidimensionality (Nasser-Abu Alhija and Wisenbaker 2006). Moreover, the use of parceling in such contexts is supported in the literature as a valid and practical approach to enhance parameter stability and reduce the influence of random item-level error (Little et al. 2002). The parcels were constructed using the item-to-construct balance method.

## Results

### Preliminary Analyses

All findings obtained within the scope of preliminary analyses are shown in Table 1. First, normality values were examined, then descriptive statistics of the variables were calculated. Additionally, correlation analysis was performed for the relationships between variables. The analysis revealed that the variables exhibited a normal distribution. Additionally, all variables were found to be statistically significant with each other. Leisure boredom had a significant positive relationship with depression (*r* = 0.236, *p* < 0.01), sensation seeking (*r* = 0.272, *p* < 0.01), and short video addiction (*r* = 0.233, *p* < 0.01). In addition, depression was positively correlated with sensation seeking (*r* = 0.234, *p* < 0.01) and short video addiction (*r* = 0.252, *p* < 0.01). Lastly, a positive significant relationship was found between sensation seeking and short video addiction (*r* = 0.249, *p* < 0.01).

**Table 1:** Sample characteristics

Variable	Frequency (*n*)	%
*Gender*		
Female	225	62.3
Male	136	37.7
*Number of Social Media Accounts*		
No social media account	15	4.2
One or two	147	40.7
Three or four	127	35.2
Five and more	72	19.9
*Duration of daily social media use*		
0–1 h	15	4.2
1–2 h	50	13.9
2–3 h	173	47.9
4+ hours	123	34.1

### Structural Equation Modeling

Following the completion of the preliminary analyses, a two-stage structural equation modeling analysis was performed using the AMOS Graphics statistical package program. Then, the significance of the mediating roles was tested using the bootstrapping method. In the first stage, the measurement model included four latent constructs (leisure boredom, depression, sensation seeking, and short video addiction) and 10 observed variables. The fit indices obtained in the measurement model are at a sufficient level: χ² (26, *N* = 361) = 86.348, *p* < 0.01; χ²/df = 2.978; CFI = 0.953; TLI = 0.927; NFI = 0.932; GFI = 0.956; IFI = 0.953; RMSEA = 0.074; SRMR = 0.050. Moreover, all factor loadings are between 0.103 and 0.922 and are significant. Although one of the observed variables had a relatively low factor loading (0.103), it was retained in the model due to its theoretical relevance to the construct.

**Fig. 1:**
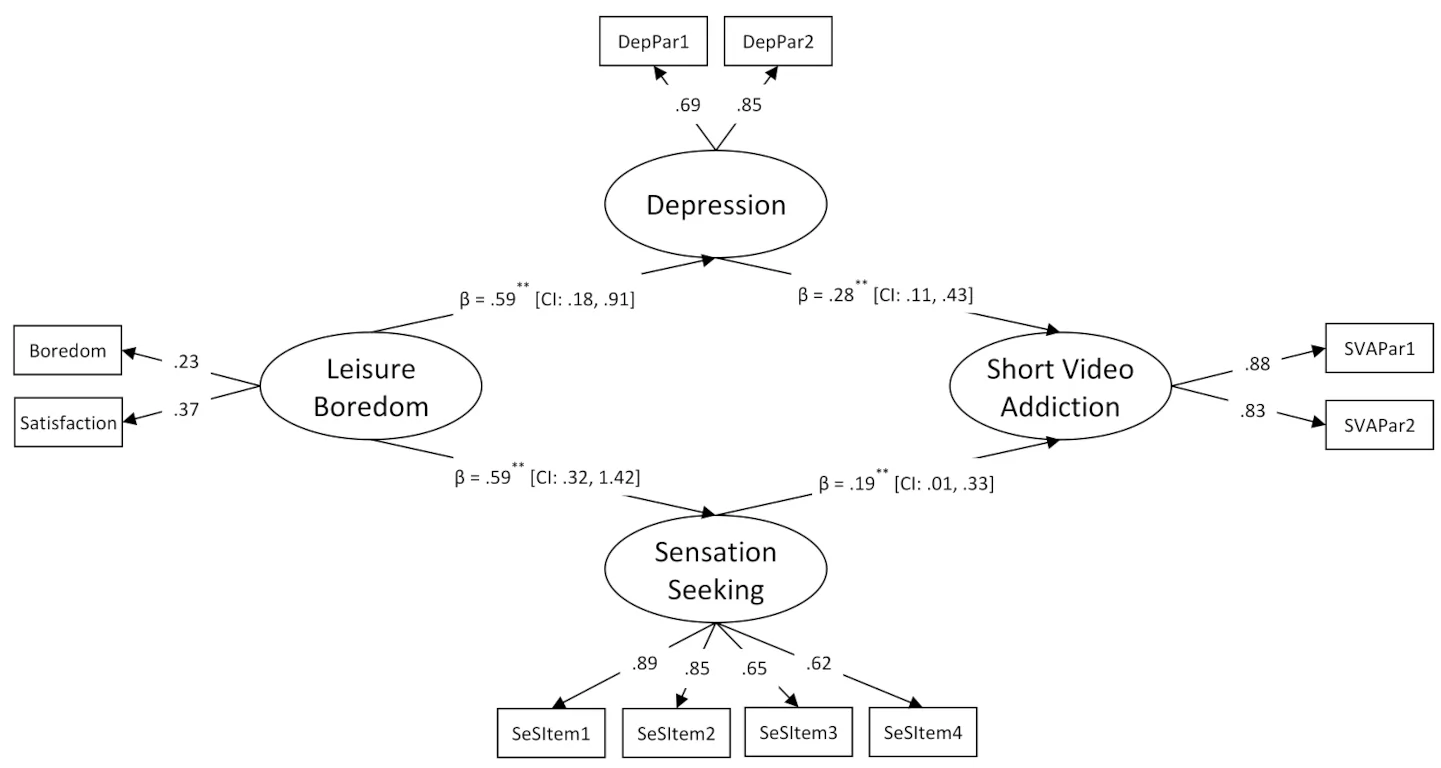
Standardized factor loading for the fully mediated structural model. *Note. N* = 361; ^**^p < 0.01; *DepPar* parcels of depression; *SeSItem* items of sensation seeking; *SVAPar* parcels of short video addiction

Following the measurement model, the structural model was tested. As a result of the analysis, it was determined that all paths in the structural model were statistically significant and the fit index values were at a sufficient level: χ² (31, *N* = 361) = 102.962, *p* < 0.01; χ²/df = 3.321; CFI = 0.941; TLI = 0.914; NFI = 0.918; GFI = 0.948; IFI = 0.942; RMSEA = 0.080; SRMR = 0.057 (see Fig. 1). This finding suggests that depression and sensation seeking multiple mediate the relationship between leisure boredom and short video addiction. An alternative model (partial mediation) was also tested. However, the direct paths from the mediators (depression and sensation seeking) to short video addiction were not statistically significant. Therefore, the full mediator model was preferred.

The significance of the mediating roles obtained was tested using the bootstrapping method. In this context, Table 2 presents the standardized coefficients for the indirect effects of the variables. As seen in Table 2, leisure boredom was found to significantly predict short video addiction through depression (bootstrap = 0.375, 95%CI = 0.109, 0.942). Furthermore, leisure boredom significantly predicted short video addiction via sensation seeking (bootstrap = 0.251, 95%CI = 0.044, 2.476). Lastly, it was revealed that leisure boredom predicted short video addiction through depression and sensation seeking (bootstrap = 0.275, 95%CI = 0.139, 0.381). This finding proves that depression and sensation seeking play a multiple and fully mediating role in the relationship between leisure boredom and short video addiction. According to Preacher and Kelley’s (2011) guidelines for evaluating indirect effect sizes, the obtained coefficients can be interpreted as moderate to large, indicating practically meaningful mediation effects.

**Table 2:** Descriptive statistics, reliabilities, and correlations for the study variables

Variable	1	2	3	4
1. Leisure Boredom	–			
2. Depression	0.236^**^	–		
3. Sensation Seeking	0.272^**^	0.234^**^	–	
4. Short Video Addiction	0.233^**^	0.252^**^	0.249^**^	–
Mean	30.110	13.706	9.526	24.299
SD	5.736	3.919	3.568	7.367
Min	10.00	6.00	4.00	10.00
Max	47.00	24.00	16.00	47.00
Skewness	-0.400	0.347	0.121	0.423
Kurtosis	0.902	-0.204	-0.872	-0.053

Note. **p < 0.01

**Table 3:** Indirect effect of serial mediation model

		95%CI	
Path	Coefficient	*LL*	*UL*
Leisure boredom → Depression → Short video addiction	0.375	0.109	0.942
Leisure boredom → Sensation seeking → Short video addiction	0.251	0.044	2.476
Total indirect effect	0.275	0.139	0.381

Note. CI confidence interval; LL lower limit; UL upper limit

## Discussion

This study examined the multiple mediating role of depression and sensation seeking in the relationship between leisure boredom and short video addiction. Within this framework, the key findings were as follows: (1) leisure boredom has an indirect effect on short video addiction, and (2) depression mediated the relationship between leisure boredom and short video addiction, and (3) sensation seeking mediated the relationship between leisure boredom and short video addiction. Consequently, the research hypotheses were confirmed.

The findings confirmed the first hypothesis of the present study (H1). Leisure boredom was found to significantly predict short video addiction among adolescents. This finding supports the theoretical framework suggesting that individuals seek stimulation to avoid low-arousal states such as boredom (Zuckerman 2014). Adolescents may turn to digital media—particularly short video platforms—as a way to cope with leisure boredom (Kardefelt-Winther 2014). However, excessive and uncontrolled use of these platforms can lead to an increased risk of addiction (Ding et al. 2024). Prior research has found that individuals who frequently experience leisure boredom tend to spend more time on short video applications (Lepp et al. 2025; Wolniewicz et al. 2020; Y. Zhang et al. 2024a, b). Therefore, both theoretical perspectives and empirical findings provide strong support for the predictive effect of leisure boredom on short video addiction.

The findings confirmed the first hypothesis of the present study (H2). Specifically, higher levels of leisure boredom were associated with an increased depression, which, in turn, predicted higher levels of short video addiction. Adolescents’ inability to structure their spare time or engage in satisfying activities may lead to experiences of leisure boredom (Weybright et al. 2015). This, in turn, may reinforce adolescents’ perceptions that they are not using their time productively, thereby fostering cognitions of inadequacy and meaninglessness (Leong and Schneller 1993). Consequently, adolescents experiencing leisure boredom are more likely to exhibit depressive symptoms (Hager et al. 2022). Leisure boredom may be a contributing factor to depression, according to the current study, which is in line with prior research (Lee and Zelman 2019; Yao et al. 2023). The findings from this study further suggest that depression may increase the risk of short video addiction among adolescents. In an attempt to cope with the negative symptoms of depression, adolescents may turn to digital media platforms (Zhang et al. 2019a, b). Such behavior may lead to excessive and uncontrolled use of short video platforms, thereby increasing the risk of developing an addiction (Zhang et al. 2024a, b). According to the Compensatory Internet Use Theory (Kardefelt-Winther 2014), negative and stressful life circumstances may drive individuals to engage more frequently in online activities. Previous research supports this view by demonstrating that depression is a significant predictor of short video addiction (Yu et al. 2024; Zhang et al. 2024a, b). This mediating role of depression highlights that the impact of leisure boredom on short video addiction does not operate directly, but rather through adolescents’ emotional vulnerability. Therefore, the existing literature supports this hypothesis (H2).

The findings confirmed the first hypothesis of the present study (H3). The findings of this study suggest that sensation seeking mediates the relationship between leisure boredom and short video addiction. Leisure boredom may lead adolescents to engage in sensation seeking behaviors (X.-C. Zhang et al. 2022). Adolescents who are unable to fill their spare time with meaningful activities may turn to sensation seeking behaviors as a way to cope with their boredom (Freund et al. 2021). According to the Optimal Arousal Theory (Zuckerman 2014), when an individual’s level of stimulation falls below a certain threshold, they may be motivated to engage in more stimulating behaviors. This finding is in line with research emphasizing the relationship between leisure boredom and sensation seeking (Lepp et al. 2025). In addition to this relationship, increased sensation seeking affects adolescents’ media use behaviors (Choi and Cho 2019). Among adolescents, sensation seeking leads to increased time spent on short video platforms, as these platforms offer content that can satisfy such needs (Huang et al. 2022). The reinforcing nature of the content on these platforms may further encourage prolonged use. As a result, adolescents may face an increased risk of developing short video addiction. Previous research has shown that higher levels of sensation seeking predict a greater risk of technology-related addiction (Meng et al. 2024; Wang et al. 2020). These findings confirm the second hypothesis (H3) of the present study.

### Limitations

There are several limitations to the research. First, as the study is cross-sectional, it is difficult to establish causal relationships between variables. Longitudinal and experimental designs in future studies would provide stronger evidence for directionality and causality. Secondly, the current study only examined the mediating roles of depression and sensation seeking. In future studies, the mediating role of different variables in this relationship can be examined. The current model can be enriched by examining the mediation roles of other variables that may be potential mediators. Thirdly, all data were collected through self-report questionnaires, which may be subject to common method bias and social desirability effects. Additionally, although the sample included adolescents from public schools that are known to serve students from diverse socio-economic backgrounds, we did not collect direct data on school type, geographic location, or SES indicators. Therefore, the generalizability of the findings should be interpreted with caution, particularly across different cultural or socio-economic contexts. Future studies may also explore moderating variables (e.g., gender, daily screen time) to extend the theoretical scope of this research. We suggest future research delve deeper into unraveling these links.

### Implications

The findings of this study have important implications for researchers and practitioners working on adolescent emotional well-being and media use habits. First, the results expand the existing literature by clarifying the psychological mechanisms—namely, depression and sensation seeking—that mediate the relationship between leisure boredom and short video addiction in adolescents. These findings contribute to the understanding of short video addiction in alignment with various theoretical perspectives. The study highlights the need for social skills-based educational programs aimed at adolescents to reduce leisure boredom and its associated negative outcomes. Additionally, the findings emphasize the necessity for parents and mental health professionals to address emotional and behavioral tendencies such as leisure boredom, depression, and sensation seeking as part of efforts to prevent short video addiction. In particular, school-based interventions that promote meaningful leisure engagement and emotional regulation strategies could play a key role in supporting adolescents at risk.

## Conclusion

This study provides empirical support for previous theoretical explanations by establishing a link between leisure boredom and short video addiction among adolescents. Given the limited number of studies examining predictors of short video addiction in adolescents, these findings can be considered a valuable contribution to this area of research. In particular, the multiple mediating roles of depression and sensation seeking offer further insight into the connection between leisure boredom and short video addiction. The results indicate that higher levels of sensation seeking intensify both depressive symptoms and sensation-seeking tendencies, ultimately leading to excessive and uncontrolled use of short video platforms among adolescents. These findings demonstrate that unstructured and unsatisfying leisure time may have a detrimental impact on adolescents’ emotional well-being. Moreover, this study provides valuable contributions to understanding the variables that influence short video addiction in adolescents. By identifying the mediating variables in the relationship between leisure boredom and short video addiction, the study contributes not only to the academic literature but also to professionals working with adolescents.

## Electronic Supplementary Material

Below is the link to the electronic supplementary material.

Supplementary Material 1

## Data Availability

Data will be available on request.

## Declarations

### Pre-Registration

This study was not pre-registered.
