# Supplementary material for: Does Leisure Boredom Predict Short Video Addiction in Adolescents?
Source: Psychiatr Q. 2025 Jun 12;97(3):729–43. doi: 10.1007/s11126-025-10172-4 (PMC13518409; doi:10.1007/s11126-025-10172-4)
Supplement: Supplementary file 1 [file 11126_2025_10172_MOESM1_ESM.docx]

**Disclosure of Interest Form**

**Conflict of Interest.** No conflict of interest exists for this manuscript for any of the author.
